# Cardiac multidetector computed tomography in infective endocarditis: a pictorial essay

**DOI:** 10.1007/s13244-014-0353-1

**Published:** 2014-09-16

**Authors:** Anaïs Grob, Franck Thuny, Chloe Villacampa, Antonin Flavian, Jean Yves Gaubert, Didier Raoult, J. P. Casalta, Gilbert Habib, Guy Moulin, Alexis Jacquier

**Affiliations:** 1 Service de Radiologie Adultes, Centre Hospitalier Universitaire Timone, Assistance Publique-Hôpitaux de Marseille, Aix-Marseille Université, Marseille, France; 2 Département de Cardiologie, Centre Hospitalier Universitaire Timone, Assistance Publique-Hôpitaux de Marseille, Aix-Marseille Université, 13005 Marseille, France; 3 URMITE, CNRS-UMR6236, Faculté de Médecine, Aix-Marseille Université, Marseille, France

**Keywords:** Infective endocarditis, Computed tomography, Multidetector, Echocardiography, Valvular damage

## Abstract

**Objectives:**

The goals of this pictorial essay are: (1) to set out a multislice computed tomography (MSCT) imaging protocol to assess infective endocarditis (IE); (2) to give an MSCT overview of valvular and peri-valvular involvement during IE; (3) to give a CT overview of septic embolism and infectious pseudoaneurysms during IE.

**Methods:**

MSCT acquisition protocols to assess IE are performed in two different phases: the first acquisition, under electrocardiography (ECG) gating, covers the cardiac structures during first-pass iodine injection; the second acquisition covers the thorax, abdomen, pelvic and cerebral regions.

**Results:**

Valvular and peri-valvular lesions during IE are: vegetation—a hypodense, homogeneous, irregular mass on a valve or endocardial structure; perforation—a defect in the leaflet; valvular aneurysm—loss of the homogenous curvature of the leaflet; valvular thickening; peri-valvular abscess; pseudoaneurysm; fistula and disinsertion of a prosthetic valve. Extra-cardiac location could involve all organs.

**Conclusions:**

MSCT can be considered as a useful complement in visualising the cardiac lesions of IE if echocardiography is inconclusive. MSCT is the only imaging modality that provides assessment of valvular and peri-valvular involvement, extra-cardiac lesions, and non-invasive evaluation of the coronary artery anatomy, simultaneously.

***Main Messages*:**

• *MSCT provides assessment of coronary anatomy, cardiac and extra-cardiac lesions.*

• *MSCT represents an alternative to echocardiography during IE.*

• *Surgical valve replacement is usually required if vegetation is >10 mm.*

• *Peri-valvular extension (abscesses, pseudoaneurysm and fistulae) required surgical treatment.*

## Introduction

Infective endocarditis (IE) is an infection of the endocardium involving the valves and adjacent cardiac structures, caused by a wide variety of bacteria and fungi. The incidence of IE is low (3-10/100,000) [1], but the in-hospital mortality rate is high, ranging between 10 % and 26 %. Despite improvement in the diagnostic and therapeutic strategies, the mortality rate due to IE has not significantly decreased since the end of the 1970s; therefore efforts must be made to explore the possibilities of new diagnostic tools such as electrocardiography (ECG-)gated multislice computed tomography (MSCT). A better knowledge of the specific lesion occurring during IE would improve and accelerate the diagnostic process, thereby avoiding fatal complications. The diagnosis of IE is based on modified Duke criteria, including clinical, biological and imaging findings (Table 1) [2]. The diagnosis of IE is often difficult, because of its variable clinical picture and complications. The main complications of the disease can affect the heart itself with destruction of the valvular apparatus, causing acute heart failure, or be systemic, leading to events such as septic embolism, haemorrhage, infectious arterial pseudoaneurysms or renal failure. The real challenge resides in improving the diagnosis of IE to initiate appropriate treatment at an early stage [3]. Echocardiography, either transthoracic echocardiography (TTE) and/or transoesophageal echocardiography (TEE), is the “gold standard” method used to assess the anatomy of the cardiac valves and peri-valvular apparatus [4]. However, the effectiveness of echocardiography may be limited by the patient’s morphology and by artefacts due to valvular calcifications or prosthetic material; furthermore, echocardiography requires a highly trained operator and results are to a certain degree operator dependent. The sensitivity of TEE is around 90 % [5], and a negative echocardiography is observed in about 15–30 % of IE cases [6]. MSCT has shown promising results in valvular and peri-valvular damage providing high-resolution anatomic information and affording multiplanar reformations [7]. MSCT also has the capacity to assess coronary artery anatomy and to diagnose peripheral embolic events in a single examination [8]. Precise, rapid assessment of cardiac and extra-cardiac lesions during IE has an impact on the choice of treatment [1]. There are few data concerning the MSCT aspects of valvular and peri-valvular involvement during IE in the literature. The goals of this pictorial essay are: (1) to set out an MSCT and CT imaging protocol to assess IE, coronary anatomy and peripheral embolic events; (2) to give an MSCT overview of valvular and peri-valvular involvement during IE; (3) to give an computed tomography (CT) overview of septic embolism and infectious pseudoaneurysms occurring during IE.

**Table 1:** Modified Duke criteria for diagnosis of infective endocarditis

Major criteria
- Typical microorganism consistent with IE from two separate blood cultures: viridans-group streptococci, *Streptococcus bovis*, HACEK group, *Staphylococcus aureus* or community-acquired enterococci, in the absence of a primary focus
OR
- Microorganisms consistent with IE from persistently positive blood cultures defined as:
. Two positive cultures of blood samples drawn >12 h apart, or
. All of three or a majority of four separate cultures of blood (with first and last sample drawn 1 h apart)
OR
- *Coxiella burnetii* detected by at least one positive blood culture or antiphase I IgG antibody titre >1:800
OR
- Positive echocardiogram showing vegetation, abscess, new valvular regurgitation or new dehiscence of prosthetic valves.
Minor criteria
• Predisposing heart disease, drug injection
• Fever: temperature >38°
• Vascular phenomena: arterial emboli, splenomegaly, mycotic aneurysm, conjunctival haemorrhages, petechiae or purpura
• Immunological phenomena: glomerulonephritis, Osler’s nodes, Roth spots or rheumatoid factor
• Microbiological evidence not fitting major criteria
Definite IE
• Two major criteria
OR
• One major and three minor criteria
OR
• Five minor criteria
Possible IE
• One major criterion and one minor criterion
OR
• Three minor criteria

## Image acquisition protocol during infective endocarditis

### Acquisition

MSCT acquisition protocols to assess IE are performed in two different phases: the first acquisition, under ECG gating, covers the cardiac structures during first pass iodine injection The parameters used for this acquisition are similar to those used for coronary assessment and have been described in detail elsewhere [9–11]. Sixty-four-slice technology is a minimum requirement for assessing valvular and peri-valvular involvement and the coronary anatomy. We restrict the use of β-blocker to patients with normal haemodynamic status. In our centre, we use a three-phase injection rationale starting with pure iodine contrast medium (60 ml for a patient around 70 kg; speed, 4 ml/s), followed by a 50 % mix of contrast medium and saline (40 ml, 4 ml/s), followed by pure saline injection (40 ml, 4 ml/s). This procedure has the advantage of enhancing the coronary tree as well as the right heart. To assess mechanical prosthetic valve motion, we perform retrospective acquisitions without ECG dose modulation. The second acquisition covers the thorax, abdomen, pelvic and cerebral regions. IE and antibiotic therapy can compromise renal function; therefore we do not use an additional iodine injection for the second acquisition; subsequently this second acquisition is performed around 60s after iodine injection. The acquisition parameters for the second acquisition are non-specific but must be adjusted to keep the radiation dose delivered to the patient as low as possible.

### Images post-processing

MSCT allows non-invasive assessment of the coronary artery anatomy prior to surgery, especially in patients at low risk of coronary artery disease and in patients with extensive IE for whom coronary angiography is associated with high risk of systemic embolism, valve or aortic wall perforation. Post-processing for coronary anatomy assessment has been described in detail elsewhere [9, 12].

The aortic and mitral valves are post-processed in the diastolic phase. The systolic phase can be used if the patients’s heart rate is above 70/min; a retrospective reconstruction around 300 ms is preferred if the heart rate is irregular. Axial images provide an overview of the data set. Multiplanar reconstructions (MPRs) are used to analyse the valves and heart chambers.

- The aortic valve is composed of an annulus, three cusps (right, left and non-coronary), and three commissures; it is analysed using three- and four-chamber views and through the aortic valve plane. Additional reconstruction can also be added in the plane of each commissure to assess the opposite cusp.
- The mitral valve [13] is composed of an annulus, two leaflets (anterior and posterior), two papillary muscles and chordae; it is analysed on four- and two-chamber views, three-chamber views (LVOT1) and LV short axis.
- The tricuspid valve has three leaflets (the anterior, septal and posterior leaflets); it is analysed on the four- and two-chamber long-axis and RV short-axis views.
- The pulmonary valve is composed of an annulus, three cusps and three commissures; it is analysed on RVOT views and along the pulmonary annulus plane.

## MSCT aspects of valvular and peri-valvular involvement in IE

The in-hospital mortality rate of patients with IE varies from 9.6 to 26 % but differs considerably from patient to patient. MSCT and CT could be useful to depict the predictors of poor outcome, offering an opportunity to adapt the choice of treatment (Table 2) [1].

**Table 2:** Predictors of poor outcome in patients with IE

Patient characteristics
Older age
Prosthetic valve IE
Insulin-dependent diabetes mellitus
*Comorbidity (e.g. previous disease, cardiovascular, renal, pulmonary)*
Presence of complications of IE
*Heart failure*
Renal failure
*Stroke*
Septic shock
*Peri-annular complication*
Valvular and peri-valvular complication
*Peri-annular complications*
Severe left-side valve regurgitation
*Low left ventricular ejection fraction*
*Sign of pulmonary hypertension*
*Large vegetation*
*Severe prosthetic dysfunction*
Premature mitral valve closure and other signs of elevated diastolic pressures

Predictors that can be depicted on MSCT and CT are in *italics*

Modified from Habib et al. [1]

### Vegetations

**Fig. 1:**
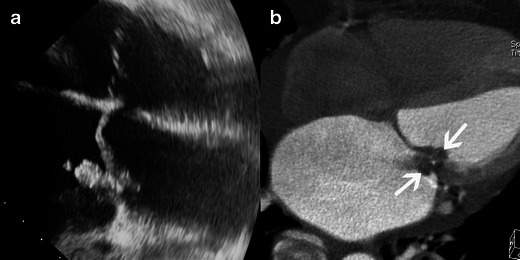
Results of echocardiography and MSCT studies in cases of mitral valve IE. Images show four-chamber views in the TEE study (**a**) and four-chamber view MSCT acquisitions with MPR reconstruction (**b**). Both TEE and MSCT show a large vegetation (*white arrow*) and destruction of the mitral valve with substantial dilatation of the left atrium and pericardial effusion

Vegetations consist of a mass of soft tissue composed of platelets, fibrin, inflammatory cells and microorganisms. They are defined on echocardiography as an oscillating or non-oscillating mass attached to a valve or other endocardial structure or on implanted intracardiac material [1]. On MSCT, vegetations can appear as a thickened valve or as irregular, homogenous, hypodense masses attached to the valve or other endocardial structures (Fig. 1, Table 3). Vegetations are mobile during the cardiac cycle and develop frequently on the atrial site of the mitral valve and on the ventricular side of the aortic valve. The migration of these vegetations explains the embolic events that occur during IE. MSCT can play a role in predicting embolic events; several factors are associated with increased risk of embolism including size and mobility, location on the mitral valve, change in size under therapy, type of microorganism (staphylococci, *Streptococcus bovis*, *Candida* spp.) previous embolism, multivalvular IE. Among these, size and mobility are the most potent independent predictors of an embolic event [1]. The risk of embolism increases with large vegetations (>10 mm) and is particularly high with very mobile and larger vegetations (>15 mm). Vegetation size is defined by the maximal length of its three spatial dimensions. The valve may require surgical replacement if the vegetation is >10 mm (Table 4) [14]. There is a strong correlation between the size of vegetations seen on MSCT and echocardiography [7]. Different studies show that 100 % of the vegetations >10 mm are diagnosed by MSCT [15]. The sensitivity of echocardiography in detecting vegetations is around 75 % for TTE and 85-90 % for TEE [16]. Feuchtner et al. [7] showed that the sensitivity and specificity of MSCT in detecting leaflet vegetation was comparable to TEE (i.e. 96 % and 97 % respectively) using intraoperative surgical findings as a standard of reference. The diagnosis may be difficult on MSCT when there are pre-existing degenerative calcified lesions of the valve and when the vegetations are less than 2 mm high [17]. Differential diagnoses for valvular vegetations are mainly prosthetic valvular thrombi and fibroelastomas.

**Table 3:** Key points of MSCT findings in IE

	MSCT Imaging findings	Advantages	Drawbacks
Vegetation	Hypodense homogeneous irregular mass on valve or endocardial structures.	- Accuracy of detection similar to echocardiography. - Vegetation size correlates on both MSCT and echocardiography.	Echocardiography is superior in detecting small vegetations.
Perforation	Defect in the leaflet.		Echocardiography is superior in detecting perforations.
Valvular aneurysm	Loss of the homogenous curvature of the leaflet		
Valvular thickening	Leaflet thickened		Difficult to assess when lesions are degenerative or calcified.
Peri-valvular abscess	Peri-valvular collection of liquid density. Thick layer of inflammatory tissue enhanced by contrast media	- Excellent detection capacity. - 3D reconstruction MSCT is superior to TEE in detecting extension to the mediastinal structures.	
Pseudoaneurysm	Abnormal cavity close to the valve enhancing concomitantly with the cardiac or aortic lumen	- Excellent detection capacity. - 3D reconstruction MSCT is superior to TEE in detecting extension to the mediastinal structures.	
Fistula	Communication between neighbouring cavities	- Excellent detection capacity. - 3D reconstruction MSCT is superior to TEE in detecting extension to the mediastinal structures.	
Prosthetic valve	Disinsertion of a prosthetic valve Vegetation on the prosthetic valve	- 3D reconstruction possible - Dynamic assessment of leaflet motion	Metallic artefacts

**Table 4:** Indication and timing of surgery for preventing embolism in left-sided native valve infective endocarditis

Indication	Timing
Aortic or mitral IE with a large vegetation (>10 mm) following one or more embolic episodes despite appropriate antibiotic therapy.	Urgent
Aortic or mitral IE with a large vegetation (>10 mm) and other predictors of subsequent complications (heart failure, persistent infection, abscess)	Urgent
Very large, isolated vegetations (>15 mm)	Urgent

Modified from Habib et al. [1]

### Valvular destruction

It is crucial to detect valvular destruction, since this can cause acute valvular regurgitation, acute heart failure and haemodynamic instability. Valvular destruction may require rapid surgical valvular replacement (Table 5)

**Table 5:** Indication and timing of surgery for heart failure in left-sided native valve infective endocarditis

Indication	Timing
Aortic or mitral IE with severe acute regurgitation or valve obstruction causing refractory pulmonary oedema or cardiogenic shock.	Emergency
Aortic or mitral IE with fistula into a cardiac chamber or pericardium causing refractory pulmonary oedema or shock	Emergency
Aortic or mitral IE with severe acute regurgitation or valve obstruction and persisting heart failure or echocardiographic signs of poor haemodynamic tolerance (early mitral closure or pulmonary hypertension)	Urgent
Aortic or mitral IE with severe regurgitation and no HF	Elective

Modified from Habib et al. [1]

#### Valvular perforation

**Fig. 2:**
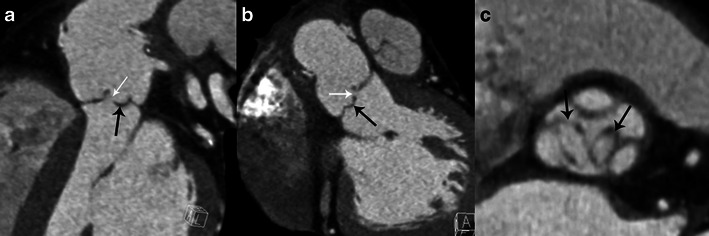
Results of MSCT studies in case of aortic valve infective endocarditis. MPR reconstructions are shown in the sagittal oblique view (**a**), coronal oblique view (**b**) and aortic valve plane view (**c**). Images show perforations at the tip of the left and non-coronary cusp (*white arrows* in **a** and **b**) and two valvular aneurysms with a ballooning of the left and non-coronary cusp (*black arrows*)

Valvular perforation is an interruption in the continuity of the valvular leaflet. A jet of valvular insufficiency visualised by colour Doppler [1] enhances the assessment of leaflet perforation on the echocardiography. On MSCT the perforation appears as a leaflet defect (Fig. 2). Several MPR reconstructions on the level of each leaflet may be required to detect perforations. Usually the perforation is located at the edge or the tip of the leaflet. Leaflet perforation is usually associated with vegetations, but may be observed alone [6]. Feuchner et al. [7] showed that TEE is more sensitive in detecting perforations than MSCT. In their study none of the four intraoperatively confirmed leaflet perforations were visualised by MSCT, whereas all four perforations were detected by echocardiography. Furthermore, Keith and al. [18] showed that three-dimensional (3D) TEE appears to be more sensitive than two-dimensional (2D) TEE in the assessment of valve perforations.

#### Valvular aneurysm

Sometimes the leaflet appears distorted with a “ballooning” effect at its extremity and loss of its homogenous curvature (Fig. 2). This may be the only structural abnormality of the leaflet and has been described as a “valvular aneurysm” [1].

#### Valvular thickening

**Fig. 3:**
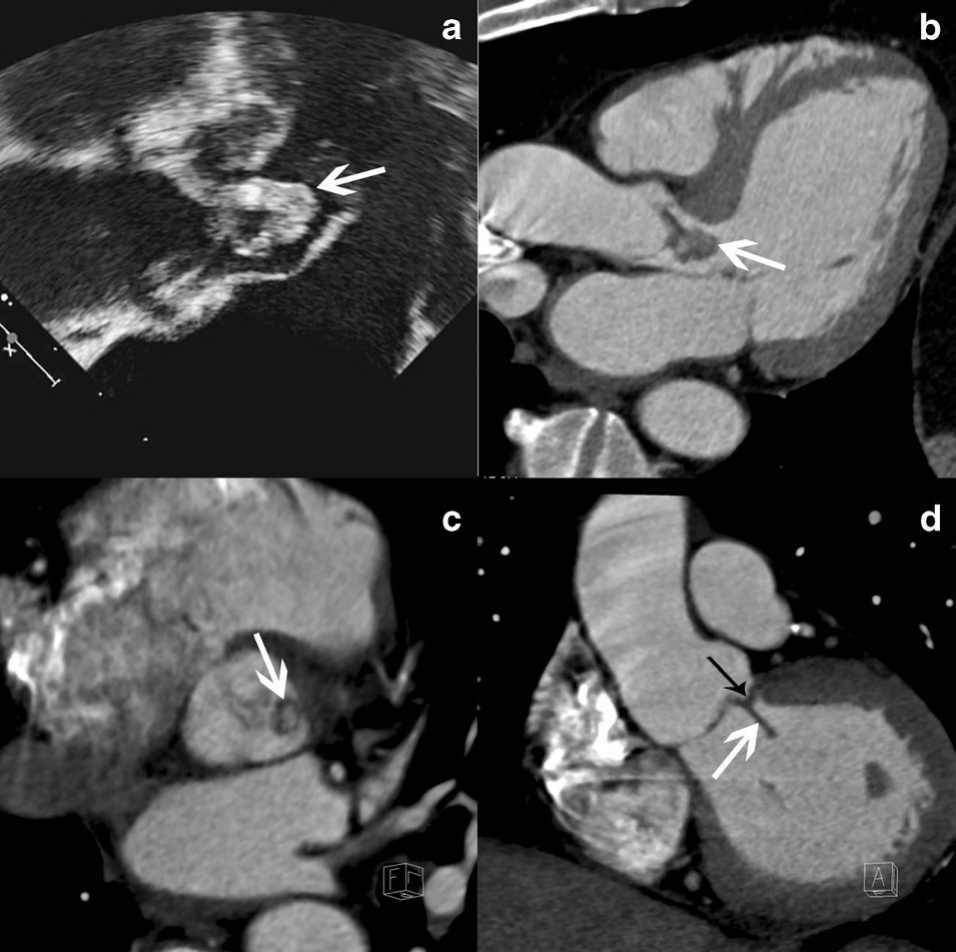
Results of echocardiography and MSCT studies in case of aortic valve infective endocarditis. Images show TEE studies, 120-degree long-axis view, at the level of the left ventricular outflow track (**a**) and MSCT acquisition with MPR reconstructions in LVOT view (**b**), aortic valve plane view (**c**) and coronal oblique view at the level of the aortic root (**d**) show a large vegetation (*white arrow*) and a valvular thickening of the right coronary aortic cusp (*black arrow*)

Vegetations can adhere along the surface of a leaflet making it appear thickened (Fig. 3). This aspect is non-specific and can also be encountered in degenerative disease.

### Peri-valvular destruction

Peri-valvular extensions such as abscesses, pseudoaneurysm and fistulae occur in 10-40 % of IE; these complications require surgical treatment (Table 6) [19]. Echocardiography is known to underestimate the presence of peri-valvular extension especially in the case of small size lesions, highly calcified valves and prosthetic valves. MSCT seemed promising in the detection of para-valvular involvement during IE [15, 20]. Feuchtner et al. showed that MSCT detected peri-valvular extension with a sensitivity and a specificity of 100 %, using surgery as the standard of reference.

**Table 6:** Indication and timing of surgery for uncontrolled infection in left-sided native valve infective endocarditis

Indication	Timing
Locally uncontrolled infection (abscess, false aneurysm, fistula, enlarging vegetation)	Urgent
Persisting fever and positive blood culture >7–10 days	Urgent
Infection caused by fungi of multi-resistant organisms	Urgent/elective

Modified from Habib et al. [1]

#### Peri-valvular abscess

**Fig. 4:**
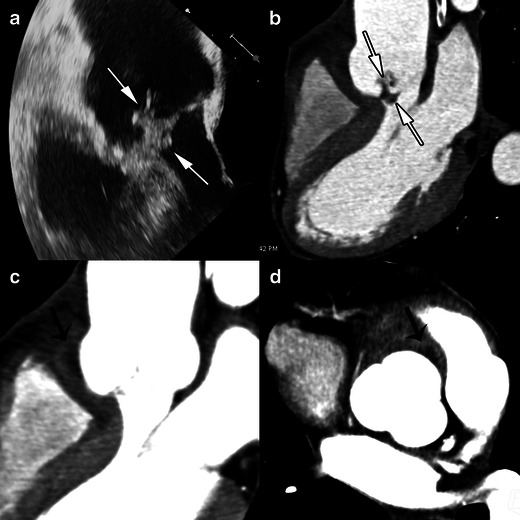
Results of echocardiography and MSCT studies in a case of aortic valve IE. The TEE study, 120-degree long-axis view (**a**) and MSCT acquisition with MPR reconstructions in the LVOT view (**b**), show a huge vegetation on the aortic valve (*white arrows*). MSCT acquisitions with MPR in the LVOT view (**c**) on the level of aortic annulus (**d**) are displayed in a narrowed imaging window, suitable for analysing para-valvular tissue, and show an abscess located in the anterior part of the aortic root (*black arrows*)

An abscess is a closed purulent collection. It is defined on echocardiography as a thickened, non-homogenous peri-valvular area with an echo-dense or echo-lucent appearance [1]. On MSCT, a peri-valvular abscess appears as a peri-valvular collection of liquid density surrounded by a thick layer of inflammatory tissue enhanced by injection of contrast medium (Fig. 4). Abscesses can infiltrate the structures surrounding the valve such as the LV myocardium or the inter-atrial septum. Abscesses are more frequently observed on the aortic valve and in prosthetic valve IE [6].

#### Pseudoaneurysm

**Fig. 5:**
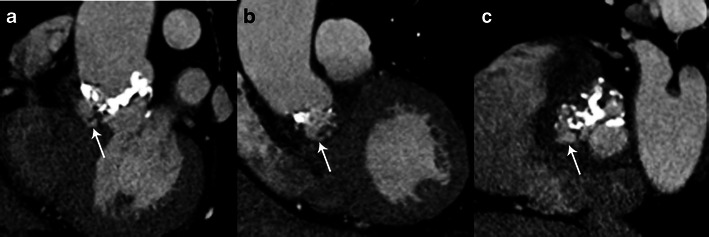
Results of MSCT studies in case of aortic valve infective endocarditis. MPR reconstructions are shown at sagittal oblique view (**a**), coronal oblique view at the level of the aortic root (**b**) and at aortic valve plane view (**c**). Images show a pseudoaneurysm with a communication between the aortic lumen and the neocavity (*white arrows*) around a degenerative, calcified, bicuspid aortic valve

Pseudoaneurysm is an abscess that has ruptured into a cardiac cavity or at the aortic root or can be due to a para-valvular leak due to the infection. On echocardiography a pseudoaneurysm is pulsatile with colour Doppler flow detected in the cavity [1]. On MSCT it appears as an abnormal cavity close to the valve, enhancing concomitantly with the cardiac or aortic lumen (Fig. 5). Communication between the cardiac cavity and pseudoaneurysm may not be detectable if the pseudoaneurysm has developed around the mitral valve.

#### Fistula

**Fig. 6:**
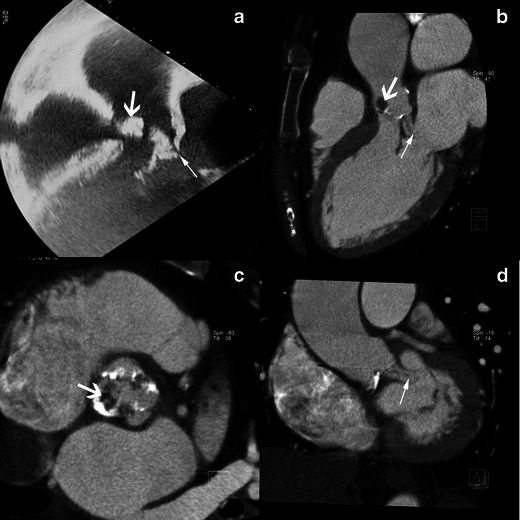
Results of echocardiography (TEE) and MSCT studies in a case of aortic valve infective endocarditis complicated by a fistula between aorta and left atrium. The TEE study is shown in the 120-degree long-axis view (**a**) and the MSCT acquisition with MPR reconstructions in the LVOT view (**b**), the aortic valve plane view (**c**), the coronal oblique view at the level of the aortic root (**d**) show a fistula between aortic root and the left atrium (*thin white arrow*) and a large vegetation on the aortic valve (*thick white arrow*)

A fistula is an abscess or a pseudoaneurysm that has ruptured into a peri-valvular cavity, creating a communication between two neighbouring cavities. It is a rare and severe complication of IE. On echocardiography a fistula is defined as a communication between two neighbouring cavities through a perforation visualised on colour Doppler [1] (Fig. 6).

### Infective endocarditis on prosthetic valves

**Fig. 7:**
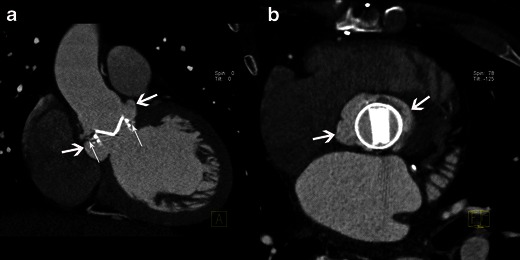
Results of MSCT study in case of infective endocarditis on a mechanical aortic valve. MPR reconstructions are shown in the coronal oblique view on the level of the aortic root (**a**) and on the aortic valve plane view (**b**). Images show disinsertion of the mechanical prosthetic valve (*thin white arrows*) and a large pseudoaneurysm around the prosthetic valve (*large white arrows*)

**Fig. 8:**
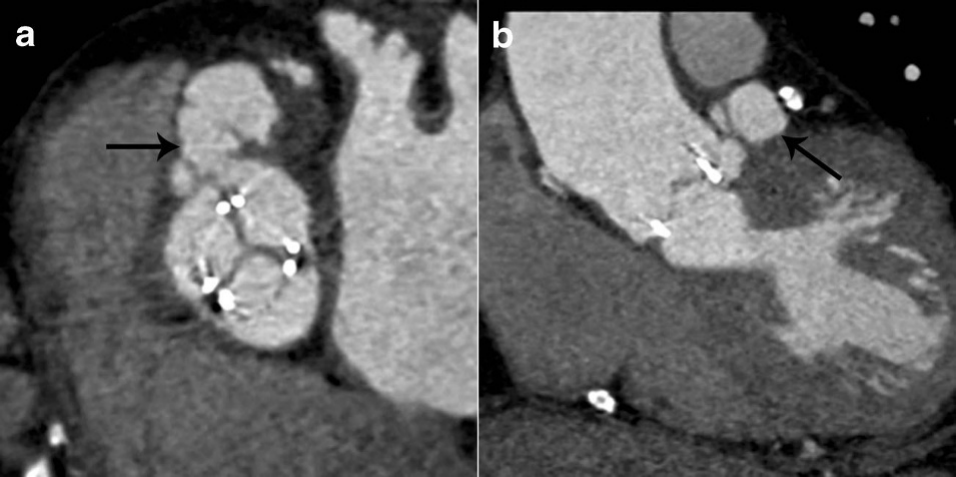
Results of MSCT studies in a case of aortic bioprosthetic valve infective endocarditis. MPR reconstructions are shown on the aortic valve plane view (**a**) and the coronal view on the level of the aortic root (**b**). Images show a large pseudoaneurysm around the bioprosthetic valve (*black arrow*)

IE is suspected on a prosthetic valve when a mass or recent, partial or complete disinsertion of a prosthetic valve is observed. It is defined on echocardiography as para-valvular regurgitation with or without rocking motion of the prosthetic valve [1]. The echocardiography is normal or inconclusive in about 30 % of cases [21]. Echocardiography is limited by acoustic shadowing, particularly when the anterior peri-arortic area is explored [22]. On MSCT, valve disinsertion presents as a pseudoaneurysm surrounding the prosthetic valve (Figs. 7 and 8). Peri-valvular infiltration is a potential finding but is usually difficult to assess due to metallic artefacts. Vegetations on prosthetic valves are also possible and appear as a mass developing on the borderline between the mobile and the fixed portion of mechanic valves or on the leaflet of bioprosthetic valves. MSCT offers high quality isovolumetric voxels affording the possibility of 3D reconstruction and dynamic assessment of leaflet motion. Fagman et al. [20] compared MSCT with TEE in 27 patients with IE on prosthetic aortic valves and found that MSCT’s diagnostic performance is comparable to TEE in the diagnosis of abscesses or dehiscence and may be a valuable complement in the preoperative evaluation of patients with a prosthetic aortic valve [20]. MSCT identified three more pseudoaneuryms that were not detected by TEE [20]. The main limitations of MSCT are artefacts caused by metal and a lack of functional assessment. Saby et al. [23] showed that adding abnormal fluorodeoxyglucose (FDG) uptake on positron emission tomography (PET)/CT around a prosthetic valve significantly increased the sensitivity of the modified Duke criteria at admission from 70 % (52–83 %) to 97 % (83–99 %); *p* = 0.008. These results open up new avenues for functional imaging in IE, and several ongoing studies should highlight the use of nuclear imaging in IE, especially in the case of prosthetic valves.

## Extra-cardiac image findings during IE with CT

Prevalence of embolic events during IE is between 20 and 50 % [24]. Embolic events and metastatic infection may involve any organ, but the central nervous system and the spleen are most frequently affected. Whole-body CT scan is appropriate for monitoring extra-cardiac manifestations/complications of IE, but to date there is no clear recommendation defining the setting in which it should be used. However, the presence of an embolic lesion diagnosed on CT scan is taken into account, when surgical treatment is envisaged to prevent embolism (Table 4).

### Neurological complications

**Fig. 9:**
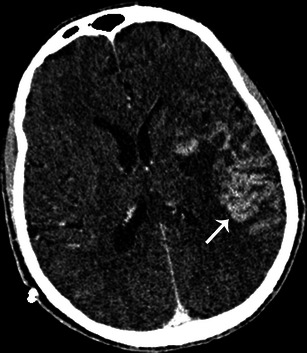
Contrast-enhanced cerebral CT following a stroke during infective endocarditis. Contrast cerebral CT shows enhancement of the cortical layer, which suggests a breakdown of the blood-brain barrier (*white arrow*)

**Fig. 10:**
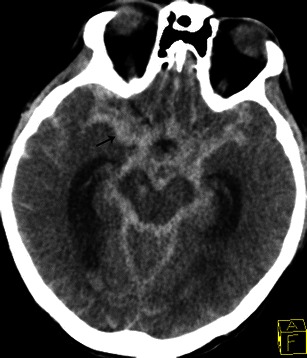
Non-contrast cerebral CT shows a spontaneous hyperdensity in the basal cistern due to subarachnoid haemorrhage (*black arrow*)

**Fig. 11:**
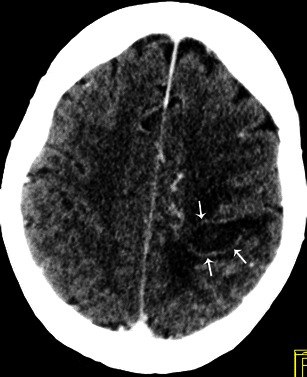
Contrast-enhanced cerebral CT shows a brain abscess as a hypodense, oval shaped lesion with peripheral enhancement (*white arrows*) and surrounding oedema in the left parietal region

In most series neurological events develop in 20-40 % of all patients with IE [24]. These are associated with a high mortality rate. Cerebral CT can be useful to detect neurological complications such as ischaemic strokes, cerebral haemorrhage and brain abscesses. Ischaemic stroke is a common complication of IE and usually presents as a hypodense area at the grey-white junction; these areas are often multiple. IV contrast can enhance the lesion, which suggests a breakdown of the blood brain barrier (Fig. 9). Intracranial haemorrhage appears as a spontaneous hyperdensity in the subarachnoid space on a non-enhanced CT (Fig. 10). Angio-CT and/or magnetic resonance imaging (MRI) should be performed to rule out mycotic aneurysms, but conventional angiography remains the gold standard. Brain abscess is a rare complication of IE. Contrast-enhanced CT shows a mixed density lesion with peripheral enhancement surrounded by oedema (Fig. 11). It is important to emphasise that MRI is more valuable in the identification of neurological complications.

### Abdominal complications

**Fig. 12:**
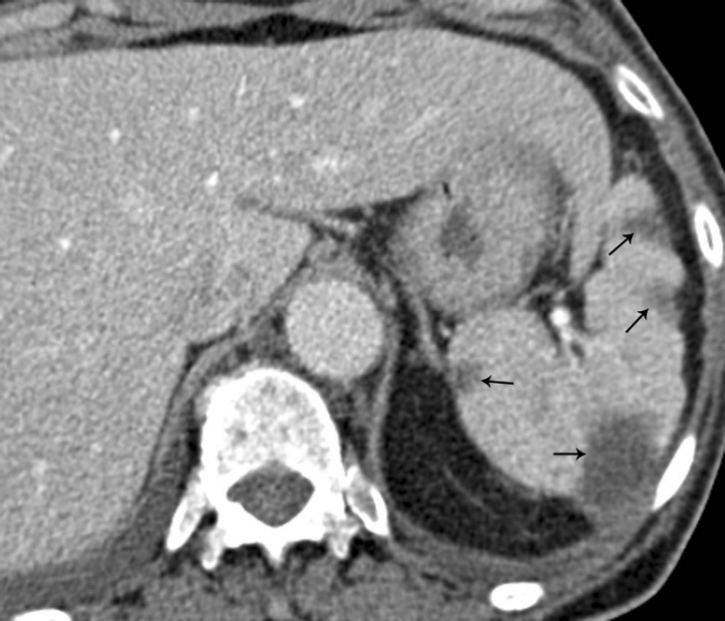
Abdominal contrast-enhanced CT shows multiple hypodense lesions in the spleen (*black arrows*). All these lesions are triangular with their broadest base on the periphery of the spleen

**Fig. 13:**
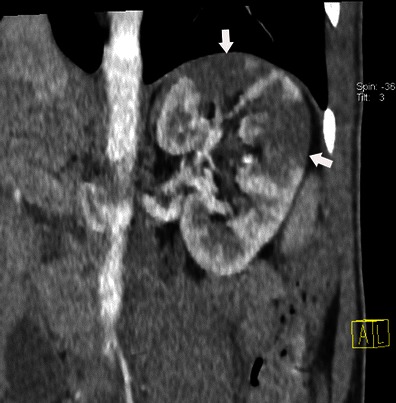
Abdominal contrast-enhanced CT shows multiple hypodense triangular areas on the left kidney. These lesions have a triangular shape with their broadest base on the periphery of the kidneys (*white arrows*)

Spleen and kidney emboli are common, estimated at 40 % for the spleen [25]. Splenic infarction appears as a triangular hypodense area on CT after contrast injection (Fig. 12). Kidney infarction showed the same pattern (Fig. 13). Abscesses can form following infarction. Abscess appears as a round-shaped mass bulging out of the normal limit of the spleen, with a non-enhanced central fluid collection and a thick, irregular wall that enhances after contrast injection. Note that liver complications are rare.

### Vascular complications

Arterial emboli occur in 22-50 % of cases. Peripheral emboli present as acute ischaemia of the extremities; IV contrast-enhancement CT in the arterial phase shows the defect in the artery.

**Fig. 14:**
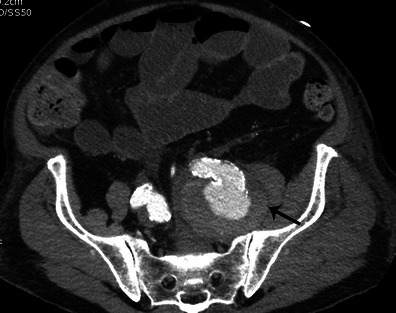
Abdominal and pelvic contrast-enhanced CT performed for the recent onset of left back pain in a patient with infective endocarditis. The axial image shows a saccular aneurysm of the ostium of the left internal iliac artery (*black arrow*)

Mycotic aneurysm is a rare complication defined as an infection of the vascular wall. Mycotic aneurysms are more frequent in the central nervous system, the abdominal aorta and the superior mesenteric artery [26]. Mycotic aneurysms appear on CT as a segmental vascular dilatation that is usually saccular (Fig. 14). The presence of peri-aneurysmal oedema and fat stranding should suggest the diagnosis [27].

### Musculoskeletal complications

**Fig. 15:**
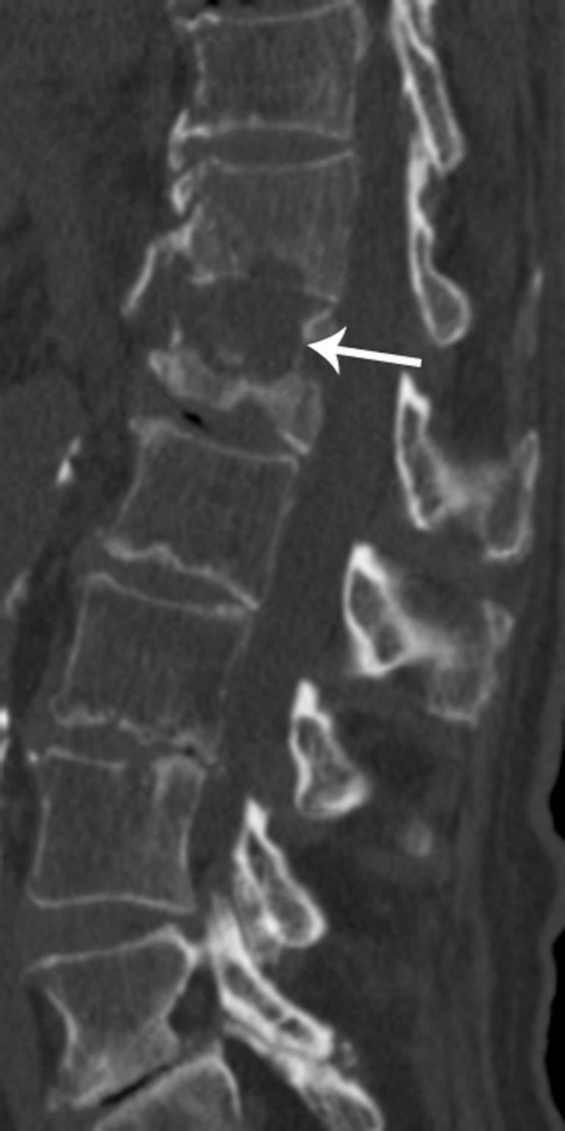
Lumbar CT-scan with sagittal reconstruction and bone windowing shows destruction of T12/L1 disk and erosion of both endplates (*white arrow*)

Manifestations of IE include spondylodiscitis, osteomyelitis and septic arthritis [26]. MRI should be preferred to assess musculoskeletal complications because CT findings can be normal in the early stages. As the disease progresses, the CT findings are: destruction of the vertebral body and fragmentation of vertebral endplates with narrowing of the disc space (Fig. 15). The presence of paraspinal collection is helpful in the diagnosis of spondylodiscitis.

### Thoracic and extracardiac complications

**Fig. 16:**
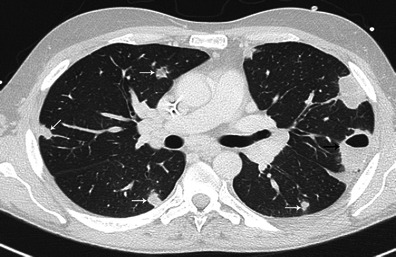
Thoracic enhanced CT shows a peripheral cavitary lesion (*black arrow*) with numerous lung nodules (*white arrows*) complicating the evolution of right side infective endocarditis

**Fig. 17:**
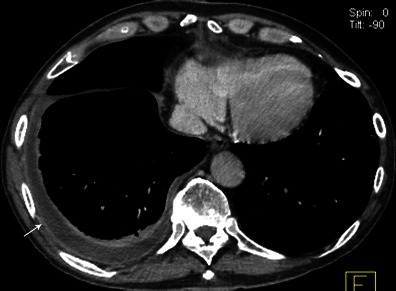
Thoracic CT shows pleural collection with enhancement of pleurae (*white arrow*)

There are almost exclusively related to right endocarditis and include pulmonary emboli, pulmonary infarction, pulmonary abscess, pleural effusion and empyema [26]. On the CT scan, pulmonary septic emboli appear as predominantly peripheral cavitary nodules, pulmonary infarcts as peripheral triangular opacities (Fig. 16). Pleural effusion and empyema are visualised as a pleural collection with enhancement of the pleurae (Fig. 17).

## Conclusions

MSCT can be considered as a useful complement in visualising the cardiac lesions of IE if echocardiography is inconclusive. MSCT is the only imaging modality that provides assessment of valvular and peri-valvular involvement, extra-cardiac lesions, and non-invasive evaluation of the coronary artery anatomy, simultaneously.
